# The GW Miracle in Many-Body Perturbation Theory for the Ionization Potential of Molecules

**DOI:** 10.3389/fchem.2021.749779

**Published:** 2021-12-21

**Authors:** Fabien Bruneval, Nike Dattani, Michiel J. van Setten

**Affiliations:** ^1^ CEA, Service de Recherches de Métallurgie Physique, Direction des Energies, Université Paris-Saclay, Paris, France; ^2^ HPQC Labs, Waterloo, ON, Canada; ^3^ IMEC, Leuven, Belgium

**Keywords:** electronic structure ab initio calculations, many-body ab initio structure, ionization potential (IP), density-functional theory (DFT), Green’s function (GF), feynman diagram expansion, coupled-cluster method, high-precision benchmarks

## Abstract

We use the GW100 benchmark set to systematically judge the quality of several perturbation theories against high-level quantum chemistry methods. First of all, we revisit the reference CCSD(T) ionization potentials for this popular benchmark set and establish a revised set of CCSD(T) results. Then, for all of these 100 molecules, we calculate the HOMO energy within second and third-order perturbation theory (PT2 and PT3), and, *GW* as post-Hartree-Fock methods. We found *GW* to be the most accurate of these three approximations for the ionization potential, by far. Going beyond *GW* by adding more diagrams is a tedious and dangerous activity: We tried to complement *GW* with second-order exchange (SOX), with second-order screened exchange (SOSEX), with interacting electron-hole pairs (*W*_TDHF_), and with a *GW* density-matrix (*γ* ^*GW*^). Only the *γ* ^*GW*^ result has a positive impact. Finally using an improved hybrid functional for the non-interacting Green’s function, considering it as a cheap way to approximate self-consistency, the accuracy of the simplest *GW* approximation improves even more. We conclude that *GW* is a miracle: Its subtle balance makes *GW* both accurate and fast.

## 1 Introduction

Many-body perturbation theory (MBPT) (Fetter and Walecka, 1971) is currently actively used to predict the excitation energies of molecules (Shirley and Martin, 1993; Grossman et al., 2001; Rostgaard et al., 2010; Blase et al., 2011; Bruneval, 2012; Körzdörfer and Marom, 2012; Ren et al., 2012; Sharifzadeh et al., 2012; Bruneval and Marques, 2013; van Setten et al., 2013; Koval et al., 2014; Govoni and Galli, 2015; van Setten et al., 2015; Blase et al., 2016; Knight et al., 2016; Kuwahara et al., 2016; Heßelmann, 2017; Maggio et al., 2017; Golze et al., 2018; Lange and Berkelbach, 2018; Wilhelm et al., 2018; Golze et al., 2019; Lewis and Berkelbach, 2019; Blase et al., 2020). The boost in the application of MBPT to molecules is being driven by the advent of physicists' methods, most noticeably the *GW* approximation (Hedin, 1965) for electron attachment and detachment energies and the Bethe-Salpeter equation (Onida et al., 1995) for neutral excitations. The present *Research Topic* acknowledges this new situation and this contribution will specifically focus on electron detachment energies.

The arrival of the physicists' methods that had been limited in their application to extended systems should not hide the fact that MBPT had been already present in chemistry for several decades, however with different approximations (Szabó and Ostlund, 1996). Indeed in the 70s, Cederbaum and coworkers explored the performance of MBPT for electron attachment and detachment energies (Cederbaum et al., 1973; Cederbaum and Niessen, 1974; Cederbaum, 1975; von Niessen et al., 1977; Cederbaum and Domcke, 1977; Cederbaum et al., 1978). Their approximations were based on a strict order-by-order expansion with respect to the electron-electron Coulomb interaction *v*. Here we name the second-order perturbation theory, PT2, and the third-order perturbation theory, PT3. Going beyond the third-order has seldom been attempted (Ortiz, 1988) for computational reasons.

The physical approximations took another path when it was realized that PT2 was producing divergent energies for the homogeneous electron gas (Mahan, 2000). It was then proposed to consider the *screened* Coulomb interaction *W* (Hedin, 1965) instead of the bare Coulomb interaction *v* as the perturbation. It turned out that the first-order correction, namely the *GW* approximation (Onida et al., 2002), was very effective for extended systems (Hanke and Sham, 1975; Godby et al., 1986; Hybertsen and Louie, 1986).

Now that the *GW* approximation has permeated chemistry, we think it is time to compare the performance in both accuracy and speed of the different approximations on a fair, unbiased basis. Fortunately, one of us has recently introduced a wide benchmark, named GW100 (van Setten et al., 2015), which consists of the ionization potentials (IP) of 100 atoms and small to medium-sized molecules. Close to twenty different codes have by now used this set to evaluate their results, and in general, when all convergence parameters are considered, the results agree well. Reference IP energies were calculated by Krause et al. (2015) via differences in the total energies calculated for the neutral and positively-charged species with the CCSD(T) approximation.

The GW100 benchmark is hence to be the boxing ring in which we want to scrutinize the quality of the different MBPT approximations (PT2, PT3, *GW*, and beyond *GW*). With *GW* we denote here the one-shot *GW* appoximation that does not include self-consistency; in the literature it is sometimes denoted as *G_0_W_0_*. *However, before doing so, we will revisit the CCSD(T) reference IPs. We observed that the set from Krause et al. (2015) is not sufficiently precise for this level of benchmarking: for instance the SO_2_ IP was more than 1 eV off the trend. We present here a complete recalculation of the CCSD(T) reference IPs for the GW100 benchmark*.

With this updated benchmark, we explain the success of the *GW* approximation for the IP of molecules: The *GW* approximation is both accurate and fast. Going beyond *GW* often worsens the result.

The article is organized as follows: In *MBPT: v-based or W-based expansions*, we recapitulate the different MBPT approximations and explain them with Goldstone-Feynman diagrams. In *CCSD(T) ionization potentials for GW100*, we set up new CCSD(T) reference values of the IPs for the GW100 benchmark set. *Benchmarking the MBPT Strategies* compares the performance of the different approximations based on a standard Hartree-Fock starting point. *MBPT From an Improved Mean-Field Starting Point* shows an attempt to approach MBPT self-consistency with tuned hybrid functionals. Finally the conclusions are drawn in *Conclusion*. Hartree atomic units are used throughout this work. The numerical values are made available as Supplemental Material, under the wide-spread machine- and human-readable JSON file format.

## 2 MBPT: *V*-Based or *W*-Based Expansions

### 2.1 Green’s Function and Self-Energy in MBPT

In MBPT, the central quantity is the one-electron Green’s function. The Green’s function describes the time-propagation of an additional particle in the electronic system: an extra electron for propagation forward in time, or a hole for propagation backward in time. The Green’s function contains a great deal of information. For instance its diagonal is the electronic density, and, most interesting for us, its poles are the ionization energies (Fetter and Walecka, 1971).

Once an approximate Green’s function *G*_0_ is known, the exact Green’s function *G* can be obtained thanks to the Dyson equation:

$$G\left(\omega \right)=G_{0}\left(\omega \right)+G_{0}\left(\omega \right)\Delta \Sigma \left(\omega \right)G\left(\omega \right),$$ (1)

where the spatial indices, later defined as *p* and *q*, have been dropped for simplicity.

The operator ΔΣ stands for the self-energy difference. It performs the humongous task of connecting *G*_0_ to *G*. If the Hartree-Fock approximation (HF) is used for *G*_0_, then ΔΣ coincides with the missing correlation part of the self-energy Σ_c_.

When a mean-field approximation is selected for *G*_0_, it can be expressed analytically:

$$G_{0\;pq}\left(\omega \right)=\delta_{pq}\frac{2}{\omega -\epsilon_{p}\pm i\eta },$$ (2)

where the factor of 2 accounts for spin, *p* and *q* are molecular orbital (MO) indices, *ϵ*_*p*_ and ± iη is a vanishing imaginary number that ensures the correct analytic behavior of *G*_*0*_. *G*_*0*_ is diagonal in the corresponding MO basis.

In practice, we make the further approximation that the self-energy difference is also diagonal in the MO basis:

$$\Delta \Sigma_{pq}\left(\omega \right)=\delta_{pq}\Delta \Sigma_{pp}\left(\omega \right).$$ (3)

This approximation is believed to be very good and is common practice in this field (Golze et al., 2019).

Recasting the Dyson Eq. 1 into

$$\left[G_{0}^{-1}\left(\omega \right)-\Delta \Sigma \left(\omega \right)\right]G\left(\omega \right)=I,$$ (4)

where *I* is the identity operator, it becomes clear that the diagonal approximation of ΔΣ will induce a diagonal approximation to *G*, since *G* and therefore also $G_{0}^{-1}$ are diagonal in the corresponding molecular basis.

Furthermore, the poles of *G* correspond to the zeroes of the term in between the brackets in Eq. 4:

$$\omega -\epsilon_{p}=\Delta \Sigma_{pp}\left(\omega \right).$$ (5)

This equation is named the quasiparticle equation and the highest zero for the *p* index that corresponds to occupied states is *ϵ*_HOMO_ = − IP. The HOMO energies reported in this work are obtained with this procedure, which is often referred to as the “graphical solution” of the quasiparticle equation (Golze et al., 2019).

We can calculate the spectral weight *Z* associated with a pole of *G* with

$$Z_{p}\left(\omega \right)={\left(1-\frac{\partial \Delta \Sigma_{pp}}{\partial \omega }\right)}^{-1}.$$ (6)

Being a weight, this quantity should range from 0 to 1 and hence $\frac{\partial \Delta \Sigma_{pp}}{\partial \omega }$ should be negative.

Note that the mean-field orbitals indexed by *p* might not be ordered properly. That is why in practice one needs to consider not only the mean-field HOMO, but also a few states below. This pathological behavior is known to occur for N_2_ for instance (von Niessen et al., 1977).

The challenge in MBPT is then to derive approximate expressions for ΔΣ that are both accurate and computationally tractable. Henceforth, we use the Goldstone-Feynman diagram representation to describe the different working approximations. The analytic expressions can be found in the cited references.

### 2.2 HF, PT2, PT3

In this Section, we follow the traditional approach in quantum chemistry for the so-called post-Hartree-Fock calculations (Szabó and Ostlund, 1996; Helgaker et al., 2000).

**FIGURE 1:**
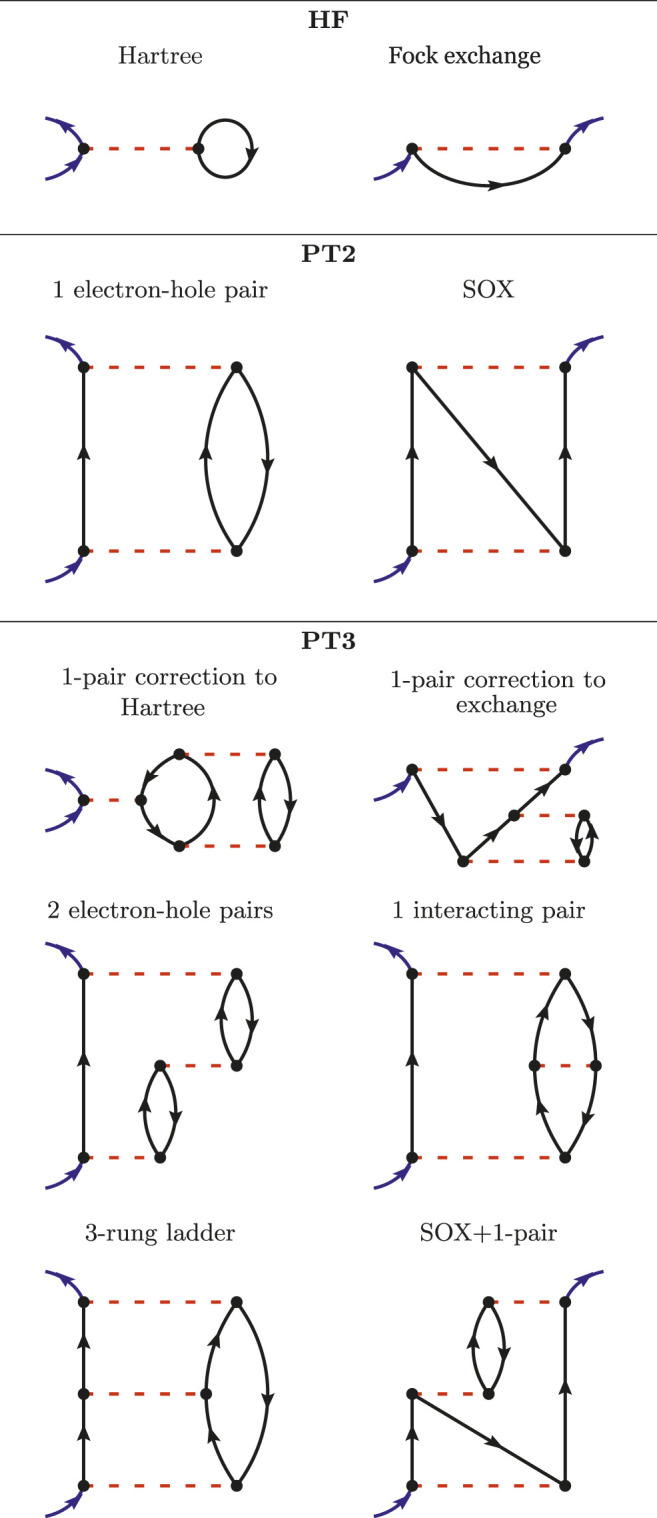
All the Goldstone-Feynman diagrams in HF **(upper panel)**, PT2 **(central panel)** and an illustrative subset of the Goldstone-Feynman diagrams in PT3 **(lower panel)**.

Let us start gradually and begin with the formulation of the HF approximation in terms of Goldstone-Feynman diagrams. In the upper panel of Figure 1, we have presented the two Goldstone-Feynman diagrams of HF: the Hartree and the Fock exchange terms. The blue arrows indicate the entry and the exit points. The black arrow is a Green’s function and the red dashed line is the bare Coulomb interaction *v*. As *v* is assumed to be instantaneous, we represent it horizontally (so that the vertical axis would be the time axis).

The Hartree diagram (upper left-hand diagram in Figure 1) translates into the following integral:

$$v_{H}\left(r\right)=\int dr^{\prime }\frac{\rho \left(r^{\prime }\right)}{\left|r-r^{\prime }\right|},$$ (7)

Where ρ(**r**) = G(**r**t, **r**t^+^) is the electronic density. From the Hartree Goldstone-Feynman diagram, we can immediately see that the Hartree potential is local in space and in time, since the entry and exit points are identical. The exchange diagram (upper right-hand diagram in Figure 1) is non-local in space, but local in time, since its entry and exit points share the same *y* coordinate.

In regular MBPT, one considers the electron-electron interaction *v* as the meaningful order parameter that will allow us to derive more and more complex approximations.

The second-order perturbation theory, PT2, considers all the possible Goldstone-Feynman diagrams having two Coulomb interactions. There are only two of those diagrams and they are drawn in the middle panel of Figure 1. The first one accounts for the propagation of an electron (or a hole) interacting with an electron-hole pair. The second one is the so-called second-order exchange (SOX). These two diagrams are still rather simple and can be found in chemistry textbooks (Szabó and Ostlund, 1996).

However, the next level, namely PT3, brings in many new terms. PT3 considers all the possible Goldstone-Feynman diagrams with three Coulomb interactions, which results in the analytic terms reported in the Appendix of Ref. (Cederbaum and Domcke, 1977). The formulas extend over three printed pages and will not be reproduced here. We will instead draw a few instructive Goldstone-Feynman diagrams in the lower panel of Figure 1. PT3 contains some static diagrams (the A-diagrams in Cederbaum’s notation), such as the two first diagrams drawn in the PT3 panel. They can be interpreted as corrections to the Hartree and Fock terms due to a correction to the density and the density-matrix. Besides these, some dynamical diagrams are displayed with two electron-hole pairs, or one interacting electron-hole pair, or a ladder diagram, etc.

The PT3 approximation had been implemented and tested by Cederbaum and coworkers (Cederbaum et al., 1973; Cederbaum and Niessen, 1974; Cederbaum, 1975; Cederbaum and Domcke, 1977; Cederbaum et al., 1978), but never applied to a systematic benchmark, to the best of our knowledge. Those authors noticed that PT3 was not fully satisfactory and proposed the rescaling of some of the terms to form a better estimate of the IP. This empirical rescaling, known as outer valence Green’s function (OVGF) or as electron propagator theory (EPT), is *not* applied here, as our focus is the MBPT itself.

Considering the huge number of terms in PT3, it is not surprising that PT4 has only rarely been used (Ortiz, 1988).

### 2.3 *W*, *GW*, SOSEX

In condensed-matter physics, it has been realized that the one-ring diagram in PT2 (See Figure 1) was producing an infinite value when evaluated for a gapless system (Mahan, 2000). A renormalized interaction was then introduced then to mitigate this problem (Baym and Kadanoff, 1961; Hedin, 1965).

**FIGURE 2:**
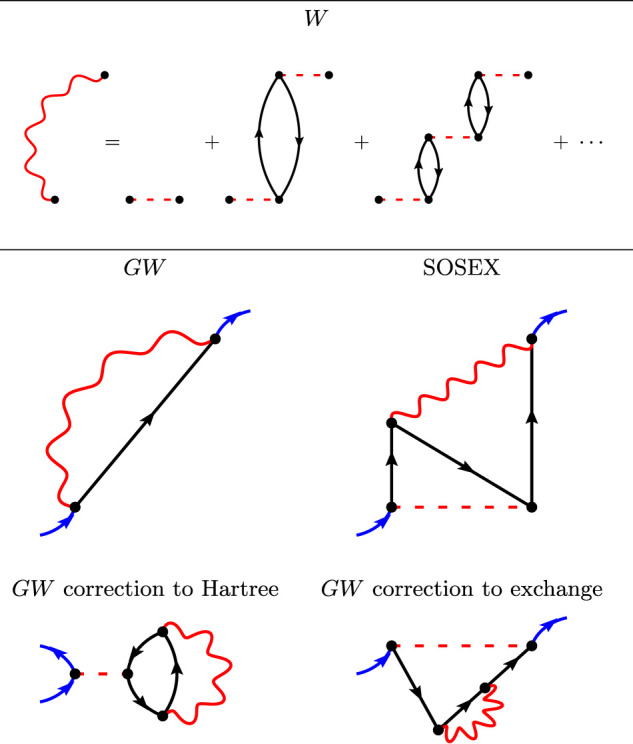
The first Goldstone-Feynman diagrams in the RPA screened Coulomb interaction *W* **(upper panel)**, the *GW* diagrams **(middle left-hand panel)**, the SOSEX diagram **(middle right-hand panel)**, and the two *γ* ^*GW*^ diagrams **(lower panel)**.

The upper panel of Figure 2 represents the screened Coulomb interaction *W* within the random-phase approximation. *W* is represented with wiggly lines that are not necessarily horizontal in the diagrams, because *W* is not instantaneous as *v* is. *W* is an infinite series of subsequent non-interacting electron-hole pairs.

There exists only one first-order diagram in *W*: the so-called *GW* approximation to the self-energy, represented in the middle left-hand panel of Figure 2. As *W* contains an infinite number of diagrams, the *GW* approximation cannot be rationalized with the *v*-based MBPT recapitulated in the previous section. Notice the similarity between the exchange diagram in HF (Figure 1) and the *GW* diagram: The Coulomb interaction has just been replaced by a non-horizontal *W* wiggly line.

This single *GW* diagram has been proven to yield very good results for the homogeneous electron gas (Hedin, 1965; Lundqvist, 1967), and for real periodic solids (Hanke and Sham, 1975; Strinati et al., 1982; Hybertsen and Louie, 1985; Godby et al., 1986). More recently, it has been realized that the same good performance is reached for molecules (Shirley and Martin, 1993; Grossman et al., 2001; Rostgaard et al., 2010; Blase et al., 2011; Bruneval, 2012; Körzdörfer and Marom, 2012; Ren et al., 2012; Sharifzadeh et al., 2012; Bruneval and Marques, 2013; van Setten et al., 2013; Koval et al., 2014; Govoni and Galli, 2015; van Setten et al., 2015; Blase et al., 2016; Knight et al., 2016; Kuwahara et al., 2016; Maggio et al., 2017; Golze et al., 2018; Lange and Berkelbach, 2018; Wilhelm et al., 2018; Golze et al., 2019; Lewis and Berkelbach, 2019; Blase et al., 2020).

Of course, the single *GW* diagram is just the first of an infinite expansion in *W*. However, the next diagrams become very complex, very quickly. They are often named “vertex corrections” in the literature. Vertex corrections appear in two different locations in Hedin’s equations (Hedin, 1965) (or equivalently in the diagrams): in *W* beyond RPA and in the self-energy itself.

Adding more diagrams in *W* would incorporate the electron-hole interaction that is present in PT3 but not in *GW*. Lewis and Berkelbach have worked on this point and showed a small effect (Lewis and Berkelbach, 2019). We will test improving *GW* along that line by using a *W* interaction calculated within time-dependent Hartree-Fock (TDHF), labeled *W*_TDHF_.

Adding more diagrams in the self-energy would incorporate the SOX diagram and more. For instance, we represent in Figure 2 the so-called second-order screened exchange (SOSEX) of Ren and coworkers (Ren et al., 2015). It is an extension to SOX that considers a screened interaction *W* together with an unscreened interaction *v*. The complete second-order diagrams with two *W* wiggly lines has been very recently considered in Ref. (Wang et al., 2021). The authors conclude that it does not bring large contributions and we will use the simpler SOSEX diagram here.

It should be added that there exist additional low-order diagrams when the Green’s function is not calculated self-consistently. Indeed, the two diagrams in the lower panel of Figure 2 are first-order diagrams in *W* that give corrections to the Hartree potential and the Fock exchange. Similar diagrams show up in PT3, however with one electron-hole pair only. These two diagrams do not appear in Hedin’s equations, because Hedin’s derivation is obtained considering the self-consistent *G*. One of us has recently studied these diagrams and highlighted a sizable effect on the IP (Bruneval, 2019a), on the electronic densities (Bruneval, 2019b), and on the total energies (Bruneval et al., 2021). We shall name these diagrams γ^*GW*^ in this work, as they only affect the one-electron reduced-density-matrix.

**FIGURE 3:**
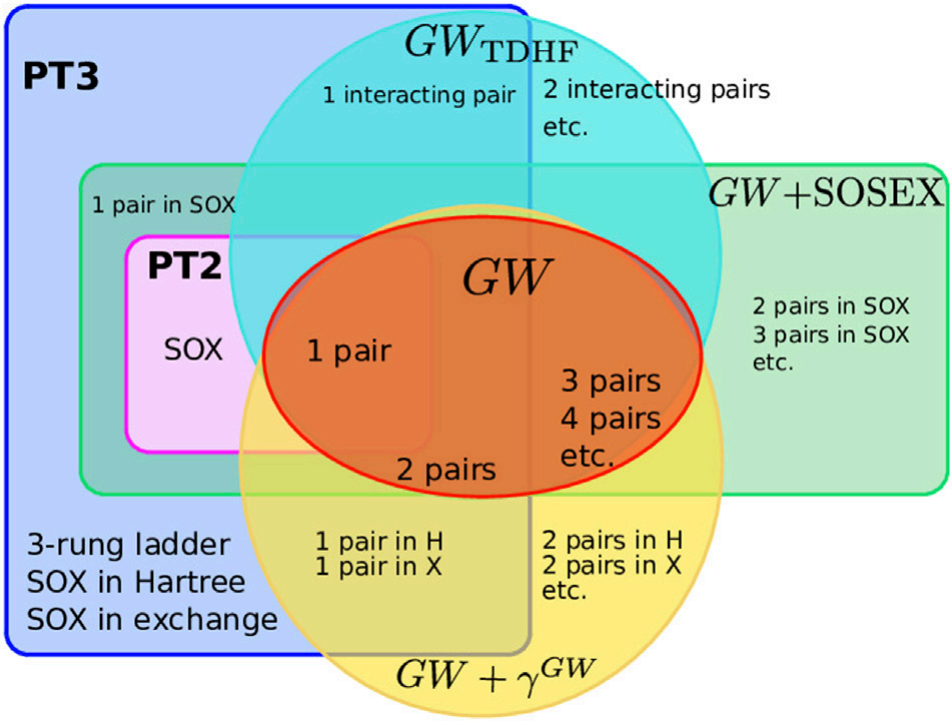
Sets of Goldstone-Feynman diagrams considered here: PT2, PT3, *GW*, *GW*_TDHF_, *GW* + SOSEX, *GW*+*γ* ^*GW*^. Some diagrams are explicitly named to give examples.

To summarize the many approximations we have presented above, Figure 3 sketches the different diagram sets used in this study. We see that PT3 contains PT2 and that *GW* has an overlap with PT2, but misses the SOX diagram. Some diagrams of *GW* are not present in PT2, nor in PT3: the *n*-pair diagrams with *n* > 2. *GW* + SOSEX entirely contains PT2, but obviously misses many diagrams of PT3. *GW*_TDDFT_ captures the 1-interacting-pair diagram of PT3 and adds the further interacting pairs. *GW* + *γ* ^*GW*^ has the 1-pair inclusion in Hartree and Fock exchange. For instance, the ladder diagram is present in PT3 only.

At this stage, there is no way to judge which approximation is best. Ideally in a perturbation theory, the more diagrams, the better. However, in MBPT, the perturbation is by no means “small” and, in our opinion, only practical calculations on trusted benchmarks are able to draw conclusions. This will be the topic of the next Sections.

## 3 CCSD(T) Ionization Potentials for GW100

### 3.1 GW100

In this work we use the set of molecules defined in the GW100 set as our boxing ring. This set came into existence first in a comparison between only three codes (van Setten et al., 2015). In the meantime the developers of many other codes have used the set to test and benchmark their implementations, both for *GW* and other computational approaches aiming at the calculation of ionization energies and electron affinities (Caruso et al., 2016; Vlček et al., 2017a; Maggio et al., 2017; Wilhelm and Hutter, 2017; Govoni and Galli, 2018; Rodrigues Pela et al., 2018; Colonna et al., 2019; Gao and Chelikowsky, 2019; Brémond et al., 2020; Förster and Visscher, 2020; Gao and Chelikowsky, 2020; Bintrim and Berkelbach, 2021; Duchemin and Blase, 2021; Förster and Visscher, 2021; Wilhelm et al., 2021). At present over a hundred data sets have appeared for the GW100 set.

The GW100 set uses established geometries and keeps them fixed for each set of calculations. In the work on the GW100 set using plane-wave basis sets in the PAW formalism using the VASP code (Maggio et al., 2017), it was noticed that for two molecules, phenol and vinyl bromide, the structure used originally was not correct. From this point the two new structures have been added to the set in order to enable comparison between sets containing only one or both versions. In this work, we use the updated geometries, so that the total number of data points is 100.

For a completely correct comparison of the molecules in the GW100 set, between codes employing different basis sets, an extrapolation to the complete basis set limits is paramount (van Setten et al., 2015; Maggio et al., 2017; Govoni and Galli, 2018). However, the use of more complete basis sets that are necessary for an extrapolation is limited by the numerical scaling of the reference CCSD(T) calculations. Fortunately, for comparisons of different methods “beyond” one-shot *GW* in codes that are based on Gaussian orbitals, this is not strictly necessary, as long as the *same* basis set is used consistently. The def2-TZVPP basis set (Weigend and Ahlrichs, 2005) has historically been used for these comparisons (Krause et al., 2015; Caruso et al., 2016). We will hence use this basis set in this work as well. In this work, we refrain from interpreting small differences below 0.1 eV that could be affected by the basis set incompleteness, so that our qualitative conclusions would be equally valid for larger basis sets.

In their work providing CCSD(T) reference values for the GW100 molecules, Krause *et al.* also used the def2-TZVPP basis set (Krause et al., 2015). Close inspection of these results however shows that in some cases large deviations with the experimental values exist and larger than one would hope for CCSD(T). Moreover, in a number of these cases the discrepancy is larger than the one between *GW* and experiment. In the present comparison we need especially accurate reference energies and since also three molecular systems of the GW100 set are missing in the data by Krause *et al.*, we start by revisiting the CCSD(T) reference set.

### 3.2 Update of the CCSD(T) Reference IP

The CCSD(T) benchmark values for the ionization potentials, which have been used by all GW100 studies up to now, were done by the authors of Ref. (Krause et al., 2015). using an unrestricted Hartree-Fock (UHF) reference and no spatial symmetry constraints. In all cases, stability analysis was done at the UHF level to ensure that the UHF solution was indeed the lowest in energy, within their convergence tolerance.

While using the lowest energy UHF solution determined via such a stability analysis, can be a very *convenient* choice, it may not lead to the most accurate CCSD(T) energy. For example, in the case of ${SO}_{2}^{+}$ , the UHF solution with the lowest energy at Hartree-Fock level, actually can lead to a higher energy at the frozen-core CCSD(T) level than a UHF solution with a higher energy at the Hartree-Fock level (see Table 1). While it is true that in general, a lower CCSD(T) energy does not necessarily mean a better one, the lowest energy in Table 1 is the closest one to our FCI (full configuration interaction, a numerically exact energy within the chosen basis set) estimates (Dattani, 2021), so the lowest CCSD(T) energy is actually the more accurate one *in this case*. Indeed, ${SO}_{2}^{+}$ was one of the worst cases in the benchmark study of Ref. (Krause et al., 2015), in terms of the disagreement between CCSD(T) and experiment for the ionization energy, and it was a case where the *GW* calculation matched the experimental ionization energy better than the “benchmark” CCSD(T) calculations did.

**TABLE 1:** Energies for ${SO}_{2}^{+}$ calculated with a default initial UHF guess in CFOUR (Matthews et al., 2020), and with the lowest-energy UHF solution. As in Ref. (Krause et al., 2015), no spatial symmetry was enforced at any time, and the number of frozen (uncorrelated) electrons was the same as for the calculations in Ref. (Krause et al., 2015).

Type of reference	SCF	CCSD(T)
UHF (default)	−546.861 914	−547.532 246
UHF (lowest)	−546.881 967	−547.488 601

In this work we have re-calculated the frozen-core CCSD(T) energies for the entire GW100 set, however we chose to use GAUSSIAN 16 (Frisch et al., 2016) (with default settings) instead of CFOUR, and the default in GAUSSIAN is an RHF (restricted Hartree-Fock) reference for all singlet species (in this paper, all neutral species), and a UHF reference for all species with a higher multiplicity (in this paper, all of the cations). This led to 46 IP values being updated with respect to Ref. (Krause et al., 2015), including the case of OCSe, for which Krause *et al.* accidentally used sulfur instead of selenium in their calculation. The most noticeable updates are SO_2_, MgO, cytosine, and uracil with changes larger than 0.4 eV.

Our revised IPs improve very much the consistency of CCSD(T) with the related method named equation-of-motion coupled-cluster (EOM-IP-CCSD). Indeed, Lange and Berkelbach (Lange and Berkelbach, 2018) have evaluated the IPs for the complete GW100 set within this approximation and found a somewhat good agreement with Krause *et al.* with an MAE of 0.09 eV. However, this correct MAE is hiding a few terrible outliers, such as SO_2_, MgO, cytosine, and uracil.

Now, comparing our updated CCSD(T) to Lange’s EOM-IP-CCSD yields not only an improved MAE of 0.06 eV, but also fixes all the mentioned outliers. The deviations between the updated CCSD(T) and EOM-IP-CCSD never exceed 0.30 eV.

As our updated CCSD(T) set very much improves the consistency across the methods and the comparison to experiment when experimental data are available, we have confidence that our updated values are a genuine improvement. We remind the Reader that all the numerical values are reported in the Supplemental Material.

## 4 Benchmarking the MBPT Strategies

A noticeable source of misunderstanding between the different MBPT flavors is the starting mean-field approximation used for the non-interacting Green’s function *G*_0_ in Eq. 2. Chemists using PT2 and PT3 typically use HF. This has several advantages: the strict order-by-order expansion is enforced and no first-order terms exist by virtue of the Brillouin theorem (Szabó and Ostlund, 1996). However, an HF *G*_0_ is maybe not the optimal Green’s function.

The physicists, quite the opposite, constantly play with the starting mean-field in order to improve the final quasiparticle energy. This strategy, sometimes named “best *G*, best *W*”, is very effective for periodic systems (Hybertsen and Louie, 1986; Aulbur et al., 1999). Indeed the HF approximation is typically not accurate for solids: the band gaps are overestimated by a lot (Silvi and Dovesi, 1988). Contrarily, *GW* based on a local density approximation (LDA) or on a semi-local approximation yields very decent results (van Schilfgaarde et al., 2006). For molecules, hybrid functionals (Bruneval and Marques, 2013) with a significant amount of Hartree-Fock exchange like BHLYP (Becke, 1993) or CAM-B3LYP (Yanai et al., 2004) are known to often produce good results.

As the discussion about the mean-field starting point can blur the conclusions, we only use an HF starting point in this Section. Discussion about an improved starting point and its connection to self-consistency is postponed to the next Section.

Henceforth, all the self-energy calculations are performed with the code MOLGW (Bruneval et al., 2016). It implements MBPT self-energies on a Gaussian-type orbital basis. It also takes advantage of the approximation of the resolution-of-the-identity (RI) (Weigend et al., 2002; Blase et al., 2011; Ren et al., 2012) with the automatic generation of the auxiliary basis set as described in Ref. (Yang et al., 2007). This technical approximation has been proven to be very accurate (Blase et al., 2016). We systematically evaluate the MBPT self-energy for the four highest occupied molecular orbitals in order to cure the possible incorrect ordering of the states in the starting mean-field approximation.

**FIGURE 4:**
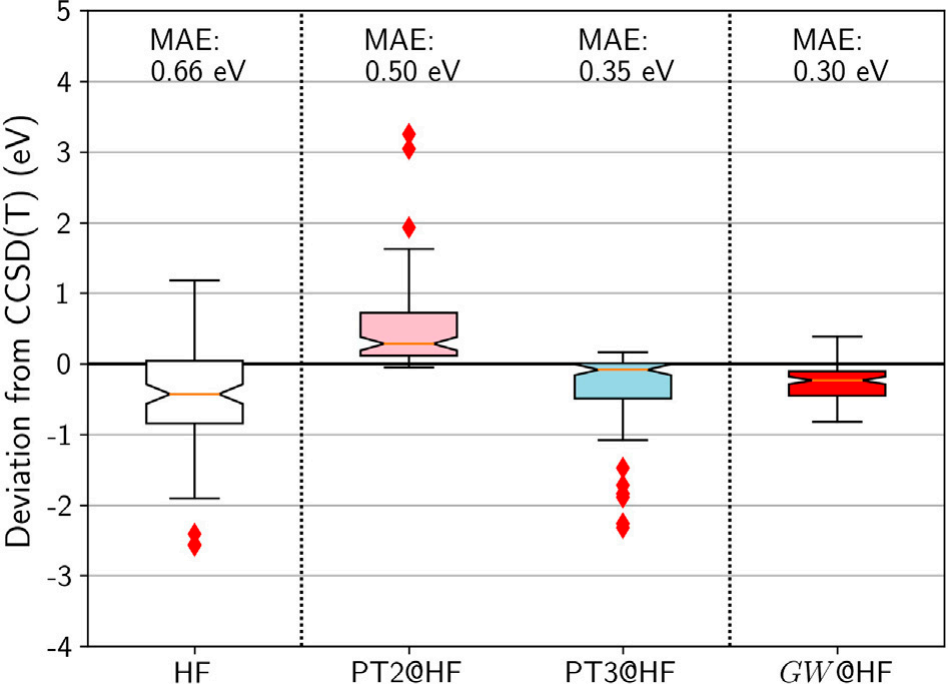
Box plots for GW100 HOMO energy errors for HF, PT2, PT3, and *GW* from an HF *G*_0_. CCSD(T) total energy differences are considered as the reference. Mean absolute errors (MAE) are also printed.

We will use box plots like in Figure 4 to summarize the error distribution of the HOMO energies with respect to CCSD(T). These plots, also known as whisker plots, report in a graphical way several relevant statistical characteristics: the median with the orange horizontal line, the first quartile with the lower box limit (25% of the distribution is below), and the last quartile with the upper box limit (75% of the distribution is below). The whiskers extend to 1.5 times the first to last quartile distance on each side. They are used to determine the so-called outliers, which are shown with the red diamonds. In addition to these box plots, we also provide the mean absolute error:

$${MAE}^{X}=\frac{1}{100}\overset{100}{\underset{i=1}{\sum }}\left|\epsilon_{HOMO,i}^{X}-\epsilon_{HOMO,i}^{CCSD\left(T\right)}\right|,$$ (8)

Where *i* runs over the 100 molecules in GW100.

### 4.1 Standard MBPT Methods: PT2, PT3, *GW*

Figure 4 shows that HF HOMO energies are too deep compared to CCSD(T), with a large spread. PT2 improves very little compared to HF: While the median is closer to zero, the mean-absolute error (MAE) remains almost as large. PT3 is a significant improvement: The median is closer to zero and the spread is reasonable. However there exists a dozen outliers with an error over 1 eV, among which the molecules containing fluorine are over-represented.

Turning to the *GW* approximation, the situation improves significantly. Not only is the MAE reduced to 0.3 eV, but also the spread is decreased. Furthermore, not a single outlier is identified in the whisker plot! It is striking to see how the computationally simpler *GW* outperforms PT3, even though PT3 contains many diagrams that *GW* does not have.

**FIGURE 5:**
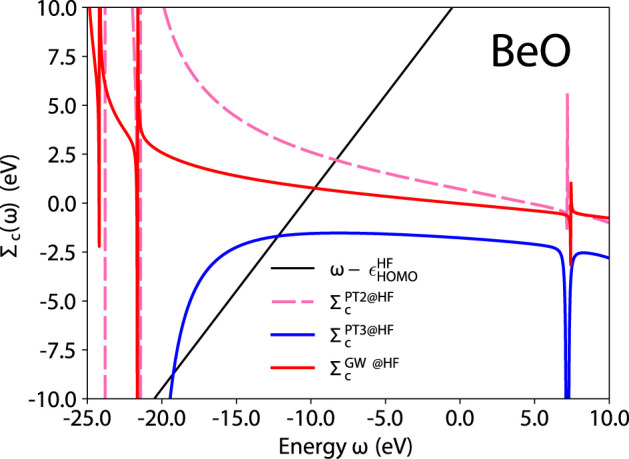
BeO quasiparticle equation graphical solution of Eq. 5 for the different self-energy approximations. HF is used for *G*_0_.

To understand some of the problems with PT3, let us analyze here in greater details the case of beryllium oxide. BeO is one of the worst failures of PT3, with a 2.26 eV deviation from CCSD(T). In Figure 5 we represent the correlation part of the self-energy expectation value (the right-hand side of Eq. 5) and the line $\omega -\epsilon_{HOMO}^{HF}$ . The intersection between these two curves defines the quasiparticle energy.

In Figure 5, we observe a pathological behavior of PT3: its derivative *∂*Σ_*c*_/*∂ω* is sometimes positive, which is not allowed for the exact self-energy. Remember that *Z* introduced in Eq. 6 is a spectral weight. A positive slope yields a nonphysical spectral weight that exceeds 1. The PT3 analytic expression contains double poles, such as the C1, D1, C6, D6 terms in the Appendix of Ref. (Cederbaum and Domcke, 1977). These terms can induce this pathological behavior. PT2 and *GW* only contain single poles as shown for *GW* in Eq. 47 of Ref. (Bruneval et al., 2016) and have the correct analytic behavior by construction.

As a conclusion, based on the GW100 IP benchmark set, *GW* is clearly the winner by knock-out on the boxing ring: It shows the best MAE, the narrower distribution of errors, and no outlier. It has, by construction, the correct analytic behavior. Furthermore, the structure of the *GW* self-energy that contains only electron-hole pairs is perfectly suited for the RI approximation. An *N* ^4^ scaling is then achieved with the contour deformation integration technique (Mejia-Rodriguez et al., 2021) and numerical methods with better scaling have also been proposed (Foerster et al., 2011; Vlček et al., 2017b; Wilhelm et al., 2018; Duchemin and Blase, 2021). In comparison, PT2 also has *N* ^4^ scaling due to the infamous “atomic orbital to molecular orbital integral transform” step and PT3 has *N* ^5^ scaling due to the quintuple MO summations (Cederbaum and Domcke, 1977).

### 4.2 Beyond *GW*

Now a legitimate question would be whether one could improve the *GW* approximation by adding some of the diagrams shown in Figure 3.

**FIGURE 6:**
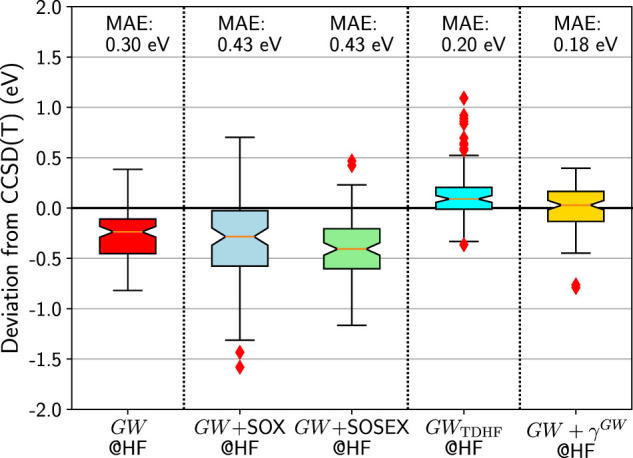
Box plots for GW100 HOMO energy errors for *GW* and beyond starting from an HF *G*_0_. CCSD(T) total energy differences are considered as the reference. Mean absolute errors (MAE) are also printed.

The simplest addition to *GW* would be to add the SOX diagram of Figure 1. This idea has already been tested by Marom *et. al.* (Marom et al., 2012) and was not successful according to them. In Figure 6, we confirm their conclusion: the results are better than PT2, but worse than *GW* alone.

Intuitively, it seems that the effect of the SOX diagram is too strong. That is why the *GW* + SOSEX proposal is appealing. The SOSEX diagram would temper the bare SOX. And this is precisely what it does: the spread of *GW* + SOSEX is narrower than that of *GW* + SOX. However, the results in Figure 6 show that the median and the MAE are still far from zero and that *GW* alone is still better.

Now let us test the possibility to incorporate the interacting electron-hole pairs, by using the TDHF screened Coulomb interaction *W*_TDHF_. This contribution gives a significant push upwards, so that the median is close to zero. Unfortunately, many outliers appear, mostly the ionic dimers of GW100, such as LiH, LiF, BeO, MgO, FH, KH. Please note that boron nitride, BN, had to be excluded from the benchmark here. Indeed the TDHF calculation failed because of a negative excitation energy. In other words, the HF self-consistent solution reached by MOLGW is not the lowest HF energy. A stability search could solve the problem (Seeger and Pople, 1977), but this implementation is not currently available in MOLGW.

Finally, we evaluate the effect of the first-order correction to the Hartree and Fock exchange terms, as depicted in Figure 2. In agreement with previous work on a smaller benchmark (Bruneval, 2019a), we observe a significant improvement over the *GW* approximation. The MAE becomes very good and the distribution is well centered around zero. The only worrying point is the existence of two outliers: TiF4 and MgO. While the TiF4 HOMO was already much too negative in *GW*@HF (−0.62 eV compared to CCSD(T)), MgO is more intriguing. It was very good with *GW* (−0.08 eV compared to CCSD(T)) and deteriorates very much with *GW* + *γ* ^*GW*^. BeO, which is chemically similar to MgO, is quite different in terms of its deviation, with a deviation of only 0.01 eV for *GW* + *γ* ^*GW*^ with respect to CCSD(T).

Of course, we did not explore all the possible combinations of diagrams beyond *GW*. However, we can state that with *GW* being already very good, it is a difficult task to improve over it. Adding diagrams may destroy the subtle balance, which makes *GW* so successful. Among all the additions we considered, only *GW* + *γ* ^*GW*^ can be considered as a systematic improvement.

## 5 MBPT From an Improved Mean-Field Starting Point

It is attractive to calculate Green’s functions self-consistently for several theoretical reasons. First, this is a systematic way to include more diagrams (Fetter and Walecka, 1971). The Green’s function lines in Figures 1, 2 would already include an infinite series of interactions. Second, Baym and Kadanoff (Baym and Kadanoff, 1961) showed that self-consistency enforces the fulfillment of several conservation laws, including the number of electrons itself.

However for practical reasons, self-consistent calculations are rarely carried out and one rather uses a one-shot approximation on top of a mean-field calculation. In the previous Section, we only used an HF mean-field for comparison reasons.

Now with the idea of approximating the self-consistent Green’s function, we can consider using an improved non-interacting Green’s function *G*_0_. For molecules, it has been identified (Bruneval, 2016; Rangel et al., 2016; Bruneval, 2019a) that hybrid functionals with boosted Hartree-Fock exchange have the best HOMO compared to CCSD(T). Then one can reasonably hope these hybrid functionals would also be good approximations to the self-consistent *G*.

**FIGURE 7:**
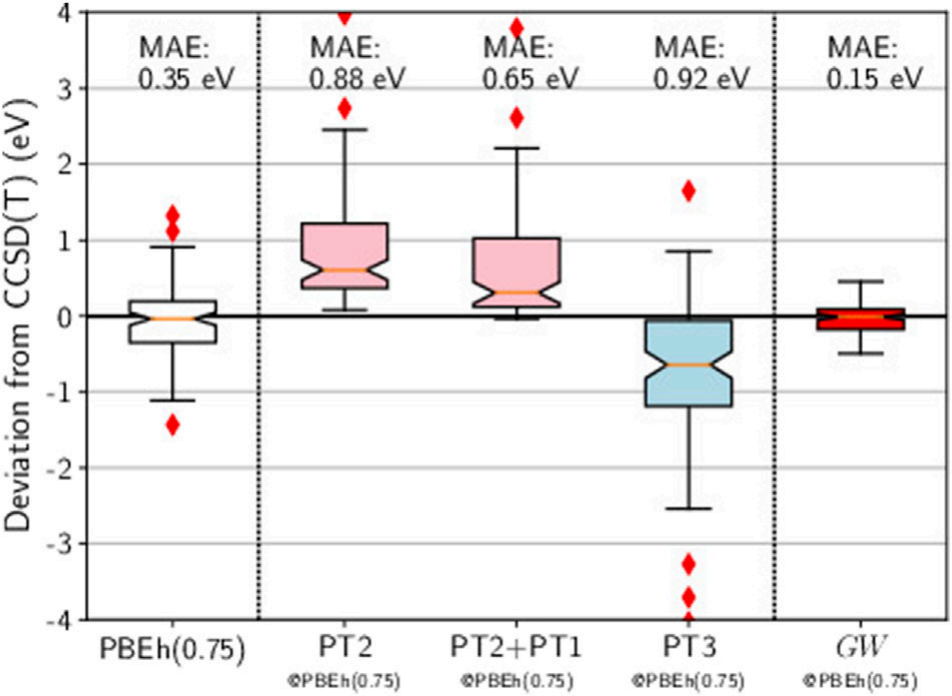
Box plots for GW100 HOMO energy errors for HF, PT2, PT2+PT1, PT3, and *GW*, starting from a PBEh(0.75) *G*_0_. CCSD(T) total energy differences are considered as the reference. Mean absolute errors (MAE) are also printed.

Here we use PBEh(0.75), a global hybrid functional which mixes the PBE exchange energy and the Hartree-Fock exchange energy in a 1:3 ratio (25% PBE, 75% Hartree-Fock). In the box plot reported in Figure 7, we show that the HOMO energies obtained with PBEh(0.75) are quite close to the CCSD(T) references: The distribution is nearly perfectly centered around zero and the MAE is reasonably low (0.35 eV).

At this point, there is a cross-road between chemistry and physics methods again. When performing a perturbation theory based on a mean-field different from HF, the Brillouin theorem breaks down (Szabó and Ostlund, 1996) and first-order terms, named PT1, appear (Ren et al., 2011). Should we include those terms? In a strict order by order expansion, the answer would be affirmative. We have tested this inclusion in the case of PT2 based on PBEh(0.75), as reported in Figure 7. Looking at the two box plots for PT2 and PT2+PT1, we conclude that the effect of the PT1 term is not significant.

Then we consider that the PBEh(0.75) Green’s function *G*_0_ is an approximation to the self-consistent *G*. As a consequence, no first-order terms appear in PT2 and some Goldstone-Feynman diagrams should be removed from the original PT3. The static diagrams (“A” diagrams in Cederbaum’s notation) are corrections to the Hartree and Fock exchange terms (See Figure 1). If PBEh(0.75) gives the correct Green’s function, it would also give the correct density and density-matrix, and then it would yield the correct Hartree and Fock exchange contributions.

Hence, Figure 7 reports the box plot of PT3 without the static diagrams. The outcome is very bad, which means that providing PT3 with a better starting point actually worsens the final result. This statement clearly advocates against PT3.

Now turning to *GW*@PBEh(0.75) in Figure 7, we obtain the best result of this study: The errors are evenly distributed around zero, no outliers are spotted, and the MAE is very low (0.15 eV). The accuracy is even better than that reached by the genuine self-consistent *GW* calculations of Caruso and coworkers (Caruso et al., 2016). It is often stated that self-consistent *GW* has quasiparticle peaks that are too weak (Holm and von Barth, 1998). We conjecture that this might be a reason why mean-field Green’s functions are superior in the end.

**FIGURE 8:**
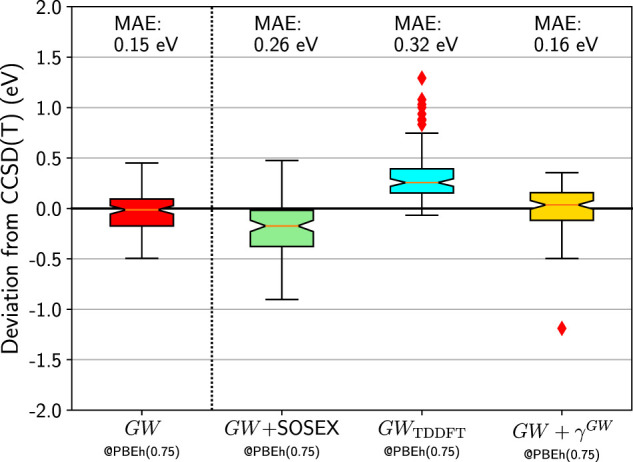
Box plots for GW100 HOMO energy errors for *GW*, *GW* + SOSEX, *GW*_TDDFT_, and *GW* + *γ* ^*GW*^ starting from a PBEh(0.75) *G*_0_. CCSD(T) total energy differences are considered as the reference. Mean absolute errors (MAE) are also printed.

Finally, we make an attempt at combining a better non-interacting Green’s function with the additional diagrams we tested in Section IVB. In Figure 8 we report the box plots for the HOMO errors with respect to CCSD(T) for *GW* + SOSEX, *GW*_TDDFT_, and *GW* + *γ* ^*GW*^ based on the PBEh(0.75) Green’s function. The *GW* + SOSEX somewhat improves compared to *GW* + SOSEX@HF. But it is still deteriorating the results compared to the simpler *GW* approximation. Next, we test *GW*_TDDFT_ where *W* was obtained from time-dependent DFT using the same functional as for *G*_0_. Again the results are disappointing.

Last, we consider *GW* + *γ* ^*GW*^. If *G*_0_ was the self-consistent *GW* Green’s function, the *γ* ^*GW*^ diagrams would vanish. Remember that the *γ* ^*GW*^ diagrams are not present in Hedin’s equations, which are obtained for a self-consistent *G*. Figure 8 shows that it is indeed the case: *GW* + *γ* ^*GW*^ is very similar to *GW*. Besides MgO, which behaves badly again, the similarity between the error distribution of *GW* and *GW* + *γ* ^*GW*^ is compelling.

## 6 Conclusion

In this study, we have conducted a comprehensive benchmark of the MBPT performance for the calculation of the IP of molecules. Our boxing ring was the GW100 set introduced by one of us (van Setten et al., 2015) a few years ago. Our reference was the CCSD(T) total energy difference, often coined as the “gold standard” in quantum chemistry. But before the competition could even start, we realized the CCSD(T) reference energies needed a thorough update. Indeed CCSD(T) energies strongly depend on the prior HF step, especially for the cations. We updated almost half of the reference IPs with respect to the existing list in Ref. (Krause et al., 2015).

Based on the same HF starting point, we evaluated the 100 HOMO energies of GW100 for PT2, PT3, *GW*, and several methods beyond *GW*. Among the classical approximations, *GW* is clearly the winner. Then our attempts to improve over *GW* by adding more diagrams have been unsuccessful, besides the *GW* + *γ* ^*GW*^ diagrams that add corrections to the Hartree and Fock exchange expectation values.

Then starting from an improved mean-field (here we chose PBEh(0.75)), deteriorates the classical approximations, PT2 and PT3. Contrarily, GW improves with a more realistic starting mean-field. Our champion is then *GW*@PBEh(0.75) with a claimed MAE of 0.15 eV.

Of course, other accurate diagrammatic techniques exist, such as the algebraic diagrammatic construction (ADC) (Schirmer et al., 1983) or equation-of-motion coupled-cluster (EOM-CC) (Lange and Berkelbach, 2018). However they do not box in the same weight class. The miracle of *GW* is the fact that its in a featherweight class: *GW*, when combined with the resolution-of-the-identify, has an attractive *N* ^4^ scaling. *GW* now routinely runs on molecular systems with several hundreds of atoms (Vlček et al., 2017b; Wilhelm et al., 2018; Bruneval et al., 2020; Duchemin and Blase, 2021).

## Data Availability

The original contributions presented in the study are included in the article/Supplementary Material, further inquiries can be directed to the corresponding author.
